# 2506: Using Amazon’s Mechanical Turk as a tool for a global survey: Lessons learned from a large-scale implementation

**DOI:** 10.1017/cts.2017.83

**Published:** 2018-05-10

**Authors:** Margaret Demment, Diana Fernandez, Dongmei Li, Susan Groth, Ann Dozier, Jack Chang, Tim Dye

**Affiliations:** University of Rochester Medical Center, Rochester, NY, USA

## Abstract

OBJECTIVES/SPECIFIC AIMS: To share lessons learned from implementing a health survey to a global sample of mTWs. METHODS/STUDY POPULATION: mTWs were paid $0.50 for taking a 15 minute survey to ascertain attitudes and intentions toward participating in genetic research. Two phases included: pilot survey targeting 7 global regions and a large-scale implementation in English in United States, India, and other countries and in Spanish in Spanish speaking countries. Administrative and descriptive information were collected and analyzed by region/country including: completions by location, demographics, time to complete, and survey satisfaction. RESULTS/ANTICIPATED RESULTS: There are 4 key lessons: (1) MTurk is fast. The US sample (n=505) accrual took <2 days and the Indian sample (n=505) took 11 days, while the response from other countries (n=118) generally exceeded 30 days. (2) Using Amazon country specification was the best way to ensure responses from specific countries and regions. (3) Demographic differences exist in mTWs between countries. For example, US mTWs were significantly more likely female (60.1%) compared with India (30.2%) and other countries (34.2%). (4) mTWs found the survey understandable/acceptable. mTWs reported high understandability and acceptability of the survey, which did not vary significantly across countries or by language. DISCUSSION/SIGNIFICANCE OF IMPACT: mTurk provides an efficient platform for survey research from diverse US and Indian samples. In other countries and in Spanish, the mTurk mechanism yielded a smaller sample more slowly but was still effective.

